# LncRNAs and Chromatin Modifications Pattern m^6^A Methylation at the Untranslated Regions of mRNAs

**DOI:** 10.3389/fgene.2022.866772

**Published:** 2022-03-17

**Authors:** Lee O. Vaasjo

**Affiliations:** ^1^ Cellular and Molecular Biology, Tulane University, New Orleans, LA, United States; ^2^ Neuroscience Program, Brain Institute, Tulane University, New Orleans, LA, United States

**Keywords:** lncRNA, M6A, histone methlyation, RNA modification, antisense lncRNA, RNA guide, UTRs

## Abstract

New roles for RNA in mediating gene expression are being discovered at an alarming rate. A broad array of pathways control patterning of N^6^-methyladenosine (m^6^A) methylation on RNA transcripts. This review comprehensively discusses long non-coding RNAs (lncRNAs) as an additional dynamic regulator of m^6^A methylation, with a focus on the untranslated regions (UTRs) of mRNAs. Although there is extensive literature describing m^6^A modification of lncRNA, the function of lncRNA in guiding m^6^A writers has not been thoroughly explored. The independent control of lncRNA expression, its heterogeneous roles in RNA metabolism, and its interactions with epigenetic machinery, alludes to their potential in dynamic patterning of m^6^A methylation. While epigenetic regulation by histone modification of H3K36me3 has been demonstrated to pattern RNA m^6^A methylation, these modifications were specific to the coding and 3′UTR regions. However, there are observations that 5′UTR m^6^A is distinct from that of the coding and 3′UTR regions, and substantial evidence supports the active regulation of 5′UTR m^6^A methylation. Consequently**,** two potential mechanisms in patterning the UTRs m^6^A methylation are discussed; (1) Anti-sense lncRNA (AS-lncRNA) can either bind directly to the UTR, or (2) act indirectly *via* recruitment of chromatin-modifying complexes to pattern m^6^A. Both pathways can guide the m^6^A writer complex, facilitate m^6^A methylation and modulate protein translation. Findings in the lncRNA-histone-m^6^A axis could potentially contribute to the discovery of new functions of lncRNAs and clarify lncRNA-m^6^A findings in translational medicine.

## Introduction

**FIGURE 1:**
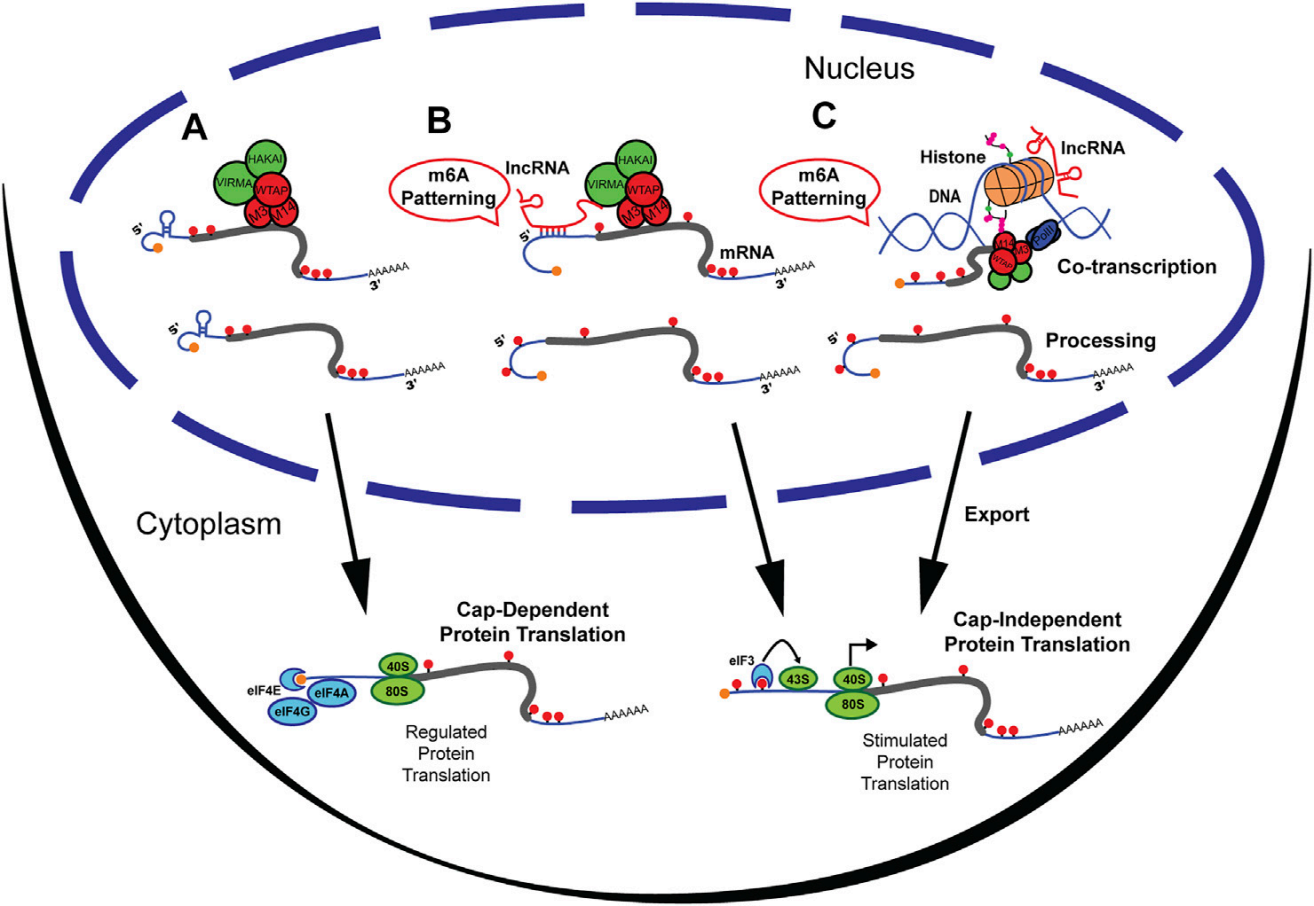
M^6^A methylation at the UTRs can be patterned by lncRNAs. **(A)** M^6^A methylation by writer complex occurring primarily at the CDS and 3′UTR of mRNA. Methylated mRNA is then exported from the nucleus and undergoes cap-dependent protein translation. **(B)** LncRNAs directly guide the m^6^A Writer complex by association with Virma to pattern the 5′UTR with m^6^A. Upon export, 5′UTR methylated mRNA undergoes Cap-independent protein translation by recruitment of eiF3 and bypassing regulatory networks. **(C)** LncRNAs can recruit histone modifying enzymes that result in m^6^A patterning. Transcripts are then exported from the nucleus and mRNAs methylated at the 5′UTR undergo Cap-independent protein translation.

RNA modifications and RNA-RNA interactions are some of the oldest biological building blocks of the cell (Schwartz, 1998; Higgs and Lehman, 2015). Long non-coding RNAs (lncRNAs) are an abundant type of non-protein-coding RNA that have diverse functions in the nucleus, including DNA organization, recruitment of histone proteins, RNA metabolism, and translational control *via* direct epigenetic interactions (Schmitz et al., 2016). LncRNAs have been described to guide DNA methylation, histone modifications, and, recently, RNA methylation (Kim et al., 2015; Marchese et al., 2017; Chen et al., 2020). While patterned by multiple mechanisms, n^6^-methyladenosine (m^6^A) methylation of RNA is the most abundant internal post-transcriptional modification and is most prevalent on the coding sequence (CDS) and 3′ untranslated region (UTR) (Meyer et al., 2012). The reversible modification of m^6^A methylation is catalyzed by “writer” proteins (Mettl3/Mettl14/WTAP) (Figure 1A), and demethylated by “erasers” (FTO/ALKBH5). M^6^A methylation has been described to be involved in alternative splicing, transport, stability of RNAs and to regulate RNA translation (B. Wu et al., 2017a; Shi H. et al., 2019). Cap-independent translation is a potent ribosome recruitment mechanism that bypasses translational control checkpoints during a rapid cellular response to environmental or physiological insults (Leppek et al., 2018). While present in low abundance, m^6^A methylation at the 5′UTR has been shown to selectively initiate cap-independent protein translation (Meyer et al., 2015; Zhou et al., 2015; Coots et al., 2017). Yet, the mechanisms that govern m^6^A patterning on the 5′UTR are poorly understood.

The 5′UTR is a critical regulator of the final product of gene expression given it can either enhance or repress the translational state of messenger RNAs (mRNAs) (Sendoel et al., 2017; Leppek et al., 2018). Since translational control is highly regulated (Silvera et al., 2010; Buffington et al., 2014), and single mRNA transcripts can persistently generate protein products (English et al., 2016), a mechanism that can tag RNAs to bypass canonical translational control is of tremendous significance. As observed in the study of the heat shock response (Meyer et al., 2015; Zhou et al., 2015), changes in m^6^A methylated 5′UTR (m^6^A 5′UTR) can alter a cell’s biological state in response to environmental cues or perturbation (Figure 1). This prompts a significant need to understand 5′UTR m^6^A patterning mechanisms. However, most studies observe a scarcity of m^6^A methylation at the 5′UTR (Fu et al., 2014). Because 5‘UTR methylation is both WTAP-independent (Schwartz et al., 2014) and Zc3h13-independent (Wen et al., 2018), this suggests that it is regulated by other sources (Meyer et al., 2012; Dominissini et al., 2013; Schwartz et al., 2014; Koranda et al., 2018). Recently, knock-out of the Mettl14/Mettl3 associated complex component Vir-like m^6^A methyltransferase associated or VIRMA (a.k.a. KIAA1429), was shown to increase the amount of 5’UTR m^6^A. This suggests that the process may be regulated by protein participants of the Mettl14/Mettl3 complex (Yue et al., 2018). Furthermore, VIRMA upregulation has been associated with tumorigenesis and seminoma cancer, consistent with aberrant gene expression profiles (Lobo et al., 2019). Studies have demonstrated that m^6^A at the 5′UTR can be altered due to biological signals such as normal development (Xiao et al., 2019), neurogenesis (Yoon et al., 2017), HIV infection (Lichinchi et al., 2016), memory formation (Widagdo et al., 2016) and stress response (J. Yu F. et al., 2018), supporting dynamic regulation of m^6^A 5′UTR. However, the mechanism by which transcript- and methylation-site specificity at the 5′UTR is controlled remains elusive (Zhao et al., 2018).

Multiple forms of regulating m^6^A methylation have been described and are frequently being discovered (Huang et al., 2020). For example, the histone modification H3K36me3 (Huang et al., 2019) was found to guide m^6^A methylation co-transcriptionally, and microRNAs (miRNAs) (Chen et al., 2015) were found to mediate binding of Mettl3 to target sites on mRNAs. Yet, both mechanisms are preferential towards m^6^A patterning of the CDS and 3′UTR. Interestingly, there are in-depth descriptions of lncRNAs that recruit chromatin modifiers, and that guide DNA methylation (Savell et al., 2016; F. Yu J. et al., 2018; Mishra and Kanduri, 2019). Non-Coding RNAs are broadly known to act as guides for RNA modifications and m^6^A is no exception; lncRNAs are now accepted as regulators of post-transcriptional modifications (Leighton and Bredy, 2018; Chen et al., 2020). Here, lncRNAs are reviewed as guides for m^6^A UTR patterning and two potential non-mutually exclusive mechanisms by which lncRNAs can dynamically control m^6^A at the UTR are discussed. In one scenario (Figure 1B), lncRNAs bind directly to the UTR of the mRNA transcripts to regulate VIRMA binding and control UTR m^6^A levels, such as lncRNA GATA3-AS (Lan et al., 2019). In the second scenario (Figure 1C), lncRNA regulate epigenetic modifications of histone subunits that ultimately pattern m^6^A on mRNA (Huang et al., 2019). This review provides an in-depth analysis of these two non-opposing mechanisms that may guide m^6^A to the 3′UTR and potentially the 5′UTR, while highlighting the cross-talk between the epigenome and the epitranscriptome.

## Co-Transcriptional Nature of m^6^A Methylation, IncRNAs and Histone Modifications

Histone modifiers, m^6^A writers, as well as hundreds of lncRNAs are thought to localize to the same subcellular nuclear compartment. However, whether these biological processes localize and can function simultaneously at a single active gene during transcription, e.g., co-transcriptionally, is a fundamental question in understanding the precise control of m^6^A methylation patterning (Perales and Bentley, 2009; Huang et al., 2020).

### M^6^A Methylation

The co-transcriptional nature of m^6^A deposition on RNA molecules was described early in the re-invigoration of the m^6^A modification field (Shi X. et al., 2019). M^6^A writers interact with transcription factors, like FoxO6 (Zong et al., 2020), with transcriptional machinery, like Poll2, along with nascent transcribed RNA (Zhou et al., 2019). Furthermore, the writer Mettl3 can bind directly with both promoter regions (Barbieri et al., 2017) and transcription start sites (TSS) (Xiao et al., 2019), and even with epigenetic machinery like histone methyltransferases (Xu et al., 2021). For example, during TGF-β pathway activation, the transcription factors SMAD2/3 promotes writer complex Mettl3, Mettl14 and WTAP activity to selectively methylate transcripts associated with cell fate specification (Bertero et al., 2018). Additionally, RNA binding proteins that bind to m^6^A sites, e.g. m^6^A “readers,” such as YTHDC1, can also interact with epigenetic machinery (Li et al., 2020). Pivotal findings have been made so far uncovering the co-transcriptional landscape of m^6^A methylation, however, these are likely only the first of many interactions with transcriptional machinery to be discovered. Overall, it is still unclear what patterning mechanisms prime the gene/transcript at the epigenetic level.

### LncRNAs in the Nucleus

LncRNAs have long been observed to interact with genomic machinery within the nucleus. These lncRNAs have been described to have direct interactions with DNA enhancer regions [e.g. Pvt1 lncRNA to MYC enhancer (Olivero et al., 2020)], transcription factors (Z. Wang et al., 2018a) (e.g., EPIC1), histones, pre-mRNA, and RNA-binding proteins within the nucleus (Yao et al., 2019). Over 120,000 species of lncRNA have been described to date (Volders et al., 2015), with thousands of lncRNAs identified within the nucleus (Frankish et al., 2019) using sequencing and fluorescent *in situ* hybridization (Cabili et al., 2015) (FISH). Specific lncRNAs demonstrate subcellular localization at nuclear speckles (Quinodoz et al., 2021), paraspeckles (Bond and Fox, 2009), and other nuclear regions such as nuclear bodies (Chujo and Hirose, 2017). Nuclear localization studies highlight how speckle-associated genomic domains tend to be rich in open-reading frames (ORFs) and highly transcriptionally active (van Steensel and Furlong, 2019). Importantly, nuclear speckles is where m^6^A methylation has been described to occur (Jia et al., 2011; Schöller et al., 2018), and where Mettl14 is known to localize *via* direct interaction with laminin-A (Zhang M. et al., 2020). While this evidence suggests nuclear speckle localizing lncRNAs could play a regulatory role in m^6^A methylation patterning, more studies are necessary to elucidate the function of lncRNAs within specific compartments of the nucleus.

### Histone Modifications and Co-Transcription

In the complex 3D environment of the nucleus, epigenetic machinery regulates gene transcription and repression. The histone proteins H2A, H2B, H3, and H4 are fundamental constituents of the nucleosome, which are modified on their N-terminal tails with reversible chromatin modifications. The best studied modifications occur on H3 and H4, which include histone acetylation (H3K27ac) and various forms of lysine methylation (H3K4me1, H3K27me3 and H3K36me3) (Zhao et al., 2021). Proteins that read these histone modifications can activate or repress DNA accessibility and bind with RNA transcription machinery (Zhao et al., 2021). Conversely, histone proteins respond to signals generated during transcription and pre-mRNA processing. The pre-mRNA processing mechanisms known to interact with histone modifications and transcription machinery include: splicing, RNA editing, 5′ end capping, and, most recently, m^6^A methylation (Bentley, 2002; Huang et al., 2020; Kan et al., 2022). Given the novelty, only a few studies have identified epigenetic-epitranscriptomic network interactions. As described in the following sections, H3K36me3 and H3K27me3 were found to bind with m^6^A writers, suggesting this new branch in the field of RNA modifications is likely to continue to expand (Huang et al., 2019; Wu et al., 2020).

## Context Dependent Changes in lncRNA Expression, 5′UTR m^6^A Patterning, and Histone Modifications

Many biological processes dynamically modulate lncRNA expression, m^6^A patterning, and the chromatin landscape (see Table 1). This review presents many of the typical physiological and pathological cell states in which all three of these epigenetic-epitranscriptomic mechanisms exhibit dynamic expression patterns. While this section lists correlational observations, many of the examples delineated here have already been described to exhibit bidirectional regulatory relationships that involve lncRNAs, histone modifications and/or m^6^A methylation.

**TABLE 1:** Correlation of regulatory dynamics in select biological and pathological states.

Cellular state	—	Mechanism	Ref
**EMT and Cancer**	lncRNA	Hundreds of lncRNAs have been associated with tumor initiation, progression, metastasis and survival rates	Du et al. (2013); Terashima et al. (2017); Wang et al. (2018b); Lv et al. (2020)
	5′UTR m^6^A	Associated with EMT transition and metastasis	Zhang et al. (2017); Lin et al. (2019); Yue et al. (2019)
	Chromatin	Histone and DNA methylation are mis-regulated in many types of cancers	Sun and Fang. (2016); Zhao et al. (2021)
**Development**	lncRNA	Over 300 positively correlated lncRNA-mRNA interactions in vertebrate development have been identified	(Devaux et al. (2015); Xiao et al. (2019); Pillay et al. (2021)
	5′UTR m^6^A	m^6^A at the 5′UTR is particularly low early in development	Batista et al. (2014); Seo et al. (2019)
	Chromatin	Histone modifications exhibit highly specific yet dynamic patterns during development	Zhang et al. (2016); Zheng et al. (2016)
**Corticogenesis**	lncRNA	Necessary for identity commitment, generation of intermediate progenitors and cellular maturation	Wu et al. (2013); Aprea and Calegari. (2015); Aprea et al. (2015); Goff et al. (2015)
	5′UTR m^6^A	Regulates cell-cycle progression of neural progenitor cells	Yoon et al. (2017)
	Chromatin	Control of progenitor renewal, generation of intermediate-progenitors and neuron migration	Mossink et al. (2021)
**Stress**	lncRNA	LncRNAs have been observed to respond to metabolite deprivation, heat-shock, and DNA damage	Audas and Lee. (2016); Pirogov et al. (2019); Cai and Jiang. (2020)
	5′UTR m^6^A	Critical in the response and regulation of stress	Zhou et al. (2015); Zhou et al. (2018); Engel et al. (2018)
	Chromatin	Precise control of histone methylation and acetylation is critical to normal physiological response to stressors	Golden et al. (2013); Wang et al. (2017a); Anderson et al. (2018)
**Learning and Memory**	lncRNA	lncRNAs can regulate activity dependent synaptic plasticity	Savell et al. (2016); Wang et al. (2017b)
	5′UTR m^6^A	m^6^A methylation is dynamically regulated during learning and is essential in memory formation	Widagdo et al. (2016); Koranda et al. (2018)
	Chromatin	Histone modifications are both critical and receptive to synaptic plasticity	Jakovcevski et al. (2015); Campbell and Wood. (2019)
**Infection**	lncRNA	Both cis- and trans acting lncRNAs can regulate host immune response during pathogen infection	Shirahama et al. (2020); Walther and Schulte. (2021)
	5′UTR m^6^A	Increase in m^6^A peaks at the 5′UTR with bacterial infection	Wu et al. (2020); Zong et al. (2020)
	Chromatin	Histone modifications are essential in host immune response or hijacked during bacterial infection	Marazzi et al. (2018)
**Reprogramming**	lncRNA	312 differentially expressed lncRNAs during cellular reprogramming	Kim et al. (2015)
	5′UTR m^6^A	Dynamic changes in 5′ UTR m^6^A in embryonic stem cells, induced pluripotent stem cells and neural stem cells	Aguilo et al. (2015); Chen et al. (2015); Zhang et al. (2020a)
	Chromatin	Histone modifications regulate and exhibit complex dynamics beginning at early stages of reprogramming	Liang et al. (2012); Onder et al. (2012); Sridharan et al. (2013)

### Changes in 5′UTR m^6^A Patterning

The dynamic mechanisms that govern the precise control of m^6^A methylation is of particular interest in the growing field of RNA modifications (Shi H. et al., 2019). Given that patterns in m^6^A can change rapidly, it has been proposed that 5′UTR m^6^A methylation may be a means of coordinated rapid response to environmental perturbation (Zhou et al., 2015). Differential and often rapid m^6^A methylation of specific transcripts has been described in multiple biological systems such as cancer, development, stress, learning and memory, infection, and cellular reprogramming (See Table 1).

The complexity of the nervous system has generated great interest in the epitranscriptome. A pioneering study of m^6^A in the brain observed dynamic changes in m^6^A levels during cortical neurogenesis and was found to be critical in mediating RNA decay during neuronal maturation (Yoon et al., 2017). In another study, the m^6^A levels at the 5′UTR of the synaptic protein DSCR1.4 increased with BDNF stimulation resulting in axon growth, confirming m^6^A involvement in central nervous system plasticity (Seo et al., 2019) and axon regeneration (Weng et al., 2018a). Interestingly, a slight increase in 5′UTR m^6^A-modified transcripts was observed within synaptosome fractions when compared to whole cell lysate (Merkurjev et al., 2018). Among the noteworthy synaptic RNAs identified by Merkurjev et al. were CaMKIIa and Shank1, that have been previously suggested to undergo non-canonical Cap-independent protein translation (Pinkstaff et al., 2001; Studtmann et al., 2014). The mammalian stress response represents another potent example of a physiological process that exhibits dynamic changes in the epitranscriptome. During stress response, changes in readers (YTHDC1), writers (Mettl3), erasers (FTO) as well as global changes in m^6^A patterns are observed. Specifically, 5′UTR m^6^A increased with response to fasting (Zhou et al., 2018), and exhibited brain region-specific dynamics in stress regulation in rodents (Engel et al., 2018). These studies fortify the notion that 5′ UTR m^6^A methylation acts as a rapid-response mechanism to physiological and environmental change.

Understanding m^6^A methylation patterns during epithelial mesenchymal transition (EMT) of oncogenes is a rapidly expanding field (Yue et al., 2019; Bera and Lewis, 2020). Increases in 5′UTR m^6^A were observed during EMT of cancer cells and during metastasis (Lin et al., 2019). The cross-talk of histone methylation and m^6^A methylation was described in great mechanistic detail and is suggested to be important during pathogen infection and the host immune response (Wu et al., 2020), as well as in playing a significant role in maintaining the pluripotency of stem cells (Huang et al., 2019). However, generally low levels of m^6^A methylation are observed during early phases of development and throughout pluripotency (Aguilo et al., 2015), but this phenomenon is poorly understood. Nevertheless, these lines of evidence support that 5′UTR m^6^A methylation exhibits context dependent patterning and coordinated rapid response.

### Dynamic lncRNA Expression

LncRNAs are well described to exhibit differential and cell-type specific expression patterns across multiple biological systems and during cell state changes including cancer (Terashima et al., 2017), stress (Carrieri et al., 2012), development (Pillay et al., 2021) and memory formation (Wang et al., 2017a) (see Table 1).

Production of anti-sense (AS) RNAs is abundant in the human brain (Mills et al., 2016). For instance, AS RNAs are integral to the epigenetic regulation of the activity dependent neuronal cFos gene during memory formation. The anti-sense FOS (AS-Fos) RNA was found to be temporally co-expressed in an activity-dependent manner with cFos mRNA. Upon cFos open reading frame activation, a transcript produced from the 3′UTR, AS-fos RNA, binds to the CpG promoter region of the Fos gene, inhibiting DNA methylation and promoting gene transcription (Savell et al., 2016). Savell et al. found AS-Fos to be essential for long-term memory formation but not short-term memory in the hippocampus during fear learning. This study alludes to the importance of temporarily precise transcriptional control by lncRNAs in the context of memory formation (Savell et al., 2016).

LncRNAs have commonly been studied in the context of stroke. One report found about 80 lncRNAs were differentially expressed during ischemic stroke, including the upregulation of the antisense lncRNA-N1LR(Z. Wu et al., 2017b). LncRNA upregulation is associated with stroke risk and recurrence (Bao et al., 2018), including antisense noncoding RNA in the INK4 locus (ANRILs) (Zhang et al., 2012). Interestingly, the expression of ANRILs is also associated with inflammation and oxidative stress (Cai and Jiang, 2020), as well as melanoma and neural tumors (Pasmant et al., 2007). This suggests lncRNA ANRILs respond to multiple cellular stressors.

Deep-sequencing studies of tumor biopsies and cancer cell lines have identified hundreds and occasionally thousands of differentially expressed lncRNAs. Among these studies, lncRNA EPIC1 (epigenetically-induced lncRNA1) was identified. EPIC1 directly interacts with the oncogene MYC and enhances MYC binding to target gene promoters resulting cell-cycle progression (Wang Y. et al., 2018). The lncRNA MEG3 is differentially expressed in during EMT transition and in multiple forms of cancer (Du et al., 2013; Terashima et al., 2017). MEG3 was found to associate with JARED2, to recruit PRC2, and induce histone H3K27 methylation on the regulatory regions of CDH1 gene. In summary, lncRNAs exhibit dynamic roles in cancer progression, many of which entail direct interactions with genes and histone modifying enzymes.

### Alterations in Histone Modifications

Epigenetic machinery is an essential core regulator and stabilizer of gene expression programs during both normal physiological and pathological states. The biological processes that regulate changes in histone modifications are heavily reviewed (Zhao et al., 2021). The epigenetic landscape is generally thought to include DNA methylation, nucleosome remodeling, 3D DNA organization, and reversible histone modifications. This review focuses on the nature of histone modifications and their potential m^6^A pattering capabilities during changes in cellular physiology.

There are hundreds of examples that describe the dynamic regulation and necessity of precise epigenetic control of chromatin remodeling during brain plasticity, stress response and development (see Table 1) (Mossink et al., 2021). Histone modifications such as H3K27ac have been extensively studied in the context of learning and memory formation (Campbell and Wood, 2019). Additionally, histone deacetylase 2 (HDAC2) is activated by glucocorticoid stress hormone and essential in regulating physiological stress response (Wang S. E. et al., 2017). Histone methyltransferases, like KMT2A and KMT2B, that regulate H3K4me are required for working memory and long-term memory formation to occur (Kerimoglu et al., 2013; Jakovcevski et al., 2015). Furthermore, increases in H3K9me2 were observed to exacerbate the anxiolytic response to withdrawal from cocaine addiction (Anderson et al., 2018). These examples highlight the capability of histone modifying enzymes to respond relatively quickly to changes in physiological state, a necessary characteristic for timely regulation of m^6^A patterning.

This review only briefly examines many types of changes in cell state that depend on the epitranscriptome and epigenome for down-stream physiological processes to occur. Importantly, for many of these, lncRNAs play essential roles. Next, many relevant mechanisms by which lncRNA act co-transcriptionally and during RNA pre-processing are discussed, as to further highlight the potential of lncRNA to pattern m^6^A methylation *via* multiple mechanisms.

## Guide Nc-RNAs in RNA Modification and Targeted AS-lncRNA Binding

Non-Coding RNAs are some of the oldest biological building blocks of the cell. This section reviews ncRNAs and lncRNAs interacting directly with RNA transcripts and as guides in RNA modification. Furthermore, given the regulatory implications of m^6^A at the 5′UTR, instances of lncRNAs binding to the untranslated regions of mRNAs are discussed. Additionally, functional categorizations of lncRNAs in terms of biogenesis and mode of action are reviewed. This section serves to contrast lncRNAs that bind with histone modifying enzymes and focuses of lncRNAs binding directly with RNA transcripts.

### NcRNAs Act as Guides in RNA Modifications

Non-coding RNAs (ncRNAs) have been studied in great depth for their ability to act as guides in RNA methylation, acetylation and pseudouridylation. These ncRNAs serve as case studies in the analysis of lncRNA-guided m^6^A methylation in the complex nuclear environment. Small nucleolar RNAs (snoRNAs) are abundant ancient ncRNAs that range between 80 and 1,000 nucleotides in length. There are at least 200 guide snoRNAs in humans, necessary for multiple post-transcriptional modifications in eukaryotic rRNAs and tRNAs(Dieci et al., 2009). SnoRNAs guide the methylation (Kiss-Laszlo, 1998; van Nues et al., 2011), acetylation (Sharma et al., 2017), and pseudouridylation (Kiss et al., 2004) of ncRNAs in order to generate functional and mature RNA species. Another example are small Cajal-body-associated RNAs (scaRNAs) that guide the post-transcriptional modification of spliceosomal small nuclear RNA (snRNAs). ScaRNA have been found to bind directly *via* RNA:RNA interactions with snRNA to guide 2’-O’methylation and pseudouridylation of the transcript (Darzacq et al., 2002). This line of evidence supports nc-RNAs and lncRNAs interacting with target RNAs in complex nuclear environments (Engreitz et al., 2016), acting on multiple RNA metabolism pathways to facilitate post-transcriptional events. However, ncRNAs binding specifically to the 5′ UTR of mRNA transcripts is significant, given the effect on translational control.

### LncRNAs can Target the UTRs

LncRNAs are well known to bind directly with target RNA transcripts causing alternative splicing, scaffolding to RNA binding proteins and change in protein translation dynamics (Yao et al., 2019). While less than 10% of developmentally active As-lncRNAs exhibit complimentary sequence overlap with 3′ UTR or 5′ UTRs of protein coding mRNA transcripts (Pillay et al., 2021), there are multiple examples of AS-ncRNAs binding to 5′UTRs. This section highlights examples of lncRNAs binding specifically to 5′UTRs.

The discovery of the antisense lncRNA for ubiquitin carboxyterminal hydrolase L1 (AS-Uchl1) was significant, given it was the first description of a lncRNA regulating protein translation at the ribosomal level (Carrieri et al., 2012). AS-Uchl1 is nuclear enriched, and upon binding with the 5′UTR of UCHL1 mRNA, both are exported to the cytoplasm. AS-Uchl1 then recruit ribosomes to initiate the translation of UCHL1 protein. Given AS-Uchl1 expression was found to be regulated by stress signaling in neurons, this alludes to fast-acting lncRNAs that can alter gene regulatory networks in response to physiological change in state (Carrieri et al., 2012).

Few studies have deciphered the mechanisms of lncRNA and 5′UTR binding. For instance, the ZEB2-AS1 was reported to bind to the 5′UTR of Zeb2 pre-mRNA after EMT. Upon binding, ZEB2-AS1 acts on the spliceosome, facilitating the retention of an internal ribosome entry site (IRES) containing intron in Zeb2 mRNA. The IRES promotes cap-independent protein translation of Zeb2 and down regulates E-cadherin (Beltran et al., 2008). Others have implicated expression of ZEB2-AS1 with shorter overall survival in patients with acute myeloid leukemia (Shi X. et al., 2019). Overall, the description of ZEB2-AS1 is a clear example of lncRNA binding to 5‘UTRs during mRNA co-transcriptional events.

These examples specifically highlight and support how antisense lncRNAs can function in different locations of the cell. AS-Uchl1 is trafficked to the cytoplasm and is an example of lncRNAs functioning outside the nucleus. In contrast, ZEB2-AS1 was an example of a lncRNA that acts within the area it was transcribed. Next, the nomenclature and functional implications of lncRNAs acting near or distant from the site of its transcription is reviewed.

### Cis- and Trans-Acting lncRNAs

**FIGURE 2:**
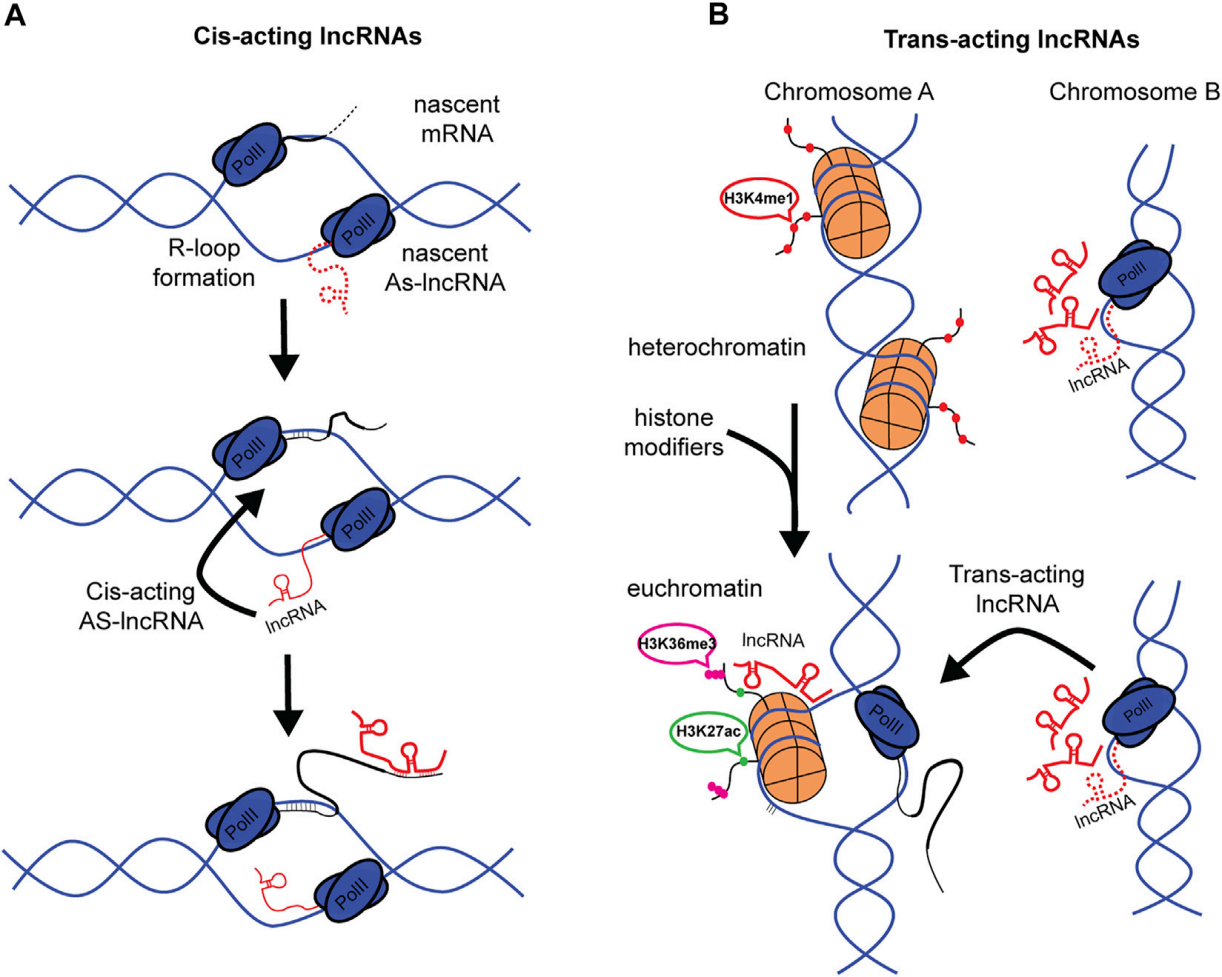
Cis- and Trans-acting lncRNAs in m^6^A patterning. **(A)** Cis-acting lncRNA can be generated by bidirectional transcription *via* R-loop formation. AS-lncRNA can then bind directly with nascent mRNA. **(B)** Representation of Trans-acting lncRNAs. Histones are shown to be repressed in Chromosome A. Change in physiological state opens chromatin to facilitate gene expression, simultaneously, lncRNAs at Chromosome B are being transcribed. LncRNAs are then trafficked to Chromosome A to guide histone modifications. (Red dots, H3K4me1. Green dots, H3K27ac. Magenta dots, H3K36me3).

The specificity of lncRNAs targeting individual mRNAs (or DNA/Chromatin) depends in part on its transcriptional origin within the genome. This review utilizes a broad classification of lncRNAs dependent on their origin and site of action; Cis-acting lncRNAs that act near the site of transcription (Figure 2A), and Trans-acting lncRNAs that act at distant sites from their locus of transcription (Figure 2B), for example, in the cytoplasm (Marchese et al., 2017; Kopp and Mendell, 2018). This classification of lncRNA facilitates interpreting the mechanism by which lncRNAs might guide m^6^A patterning, given the co-transcriptional nature of m^6^A methylation and known nuclear functions in RNA binding of distinct lncRNAs.

Cis-acting lncRNAs, or cis-antisense lncRNAs, are well known to function in gene regulation. These can be generated in a variety of ways, including bi-directional transcription during R-Loop formation (Tan-Wong et al., 2019) or presence of bi-directional promoters (Uesaka et al., 2014) (Figure 2A). These local lncRNAs are quite stable and exhibit long half-lives, with an average of 4.8 h, many exceeding 12 h, though of less duration than the mRNAs they regulate (Tani et al., 2015). Most studies agree that AS-lncRNAs mostly localize, and likely function, near their transcriptional loci. Some estimates suggest around 93% of nuclear lncRNAs are Cis-acting lncRNAs (Quinodoz et al., 2021). Given the anti-sense nature of cis-acting AS-lncRNAs, the long half-life, and the immediate proximity to target mRNAs, these AS-lncRNAs make suitable candidates as direct binding partners with the UTR and guides of m^6^A writer machinery. This hypothesis is supported by the observation that GATA3-AS lncRNA binds with GATA3 mRNA to regulate m^6^A patterning (Lan et al., 2019).

Trans-acting lncRNAs, in contrast to cis-acting lncRNAs, function at distant nuclear or cytoplasmic sites from their transcriptional loci of origin (Figure 2B). Common examples of trans-acting lncRNAs might be transcribed from pseudogenes (Muro and Andrade-Navarro, 2010; Johnsson et al., 2013) and large intergenic non-coding RNAs (lincRNAs) (Guttman et al., 2011). Trans-acting lncRNAs are known to interact with epigenetic machinery (Zhao et al., 2010), and it is this involvement in chromatin remodeling that is likely to contribute to a trans-acting pathway that alters UTR methylation patterns. This proposal is enticing, given that trans-acting lncRNAs can affect multiple gene/mRNA species through “multi-way contract” with histone remodeling complexes. This classification of lncRNAs provides insight into how different, sometimes parallel pathways might converge on RNA expression mechanisms.

## LncRNAs, Chromatin Remodeling and m^6^A Methylation Suggests Epigenetic Cross-Talk

### Examples of lncRNAs in m^6^A Dynamics

**FIGURE 3:**
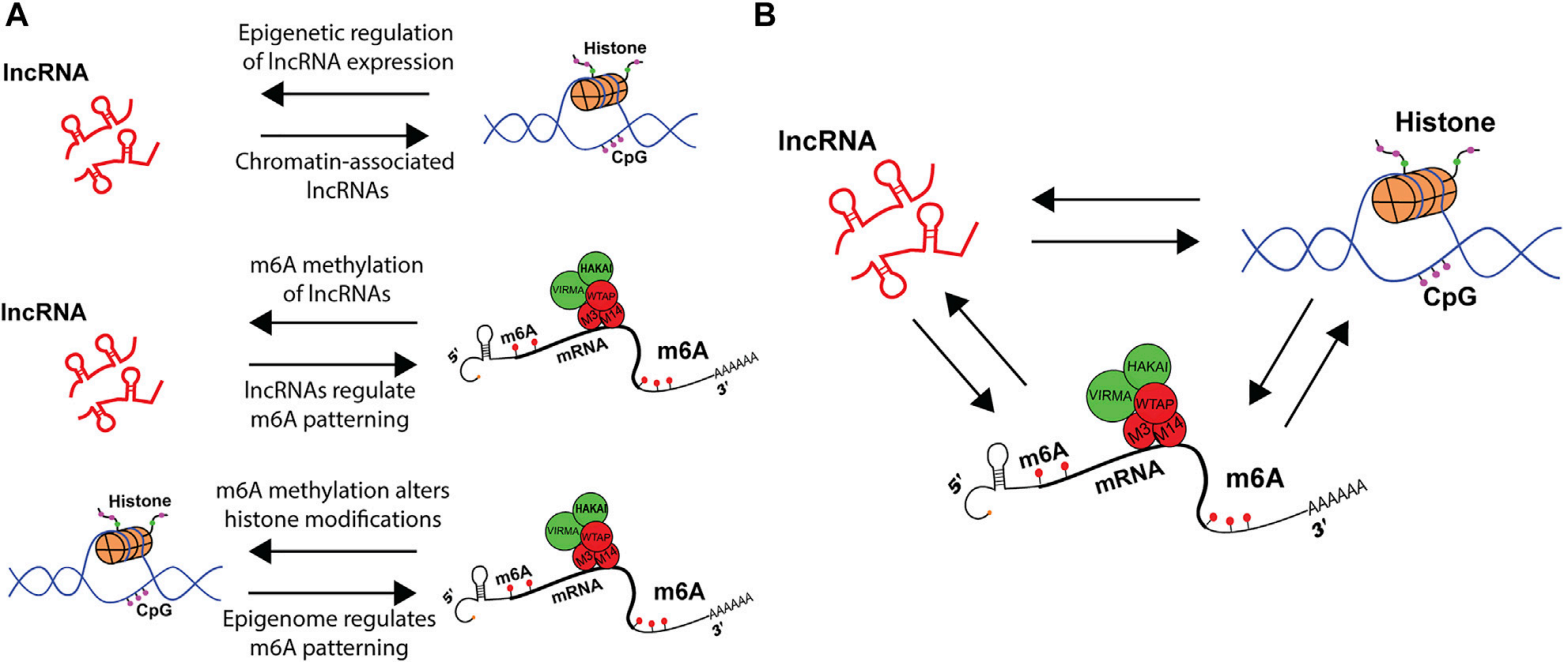
Epigenetic crosstalk among lncRNAs, histones and m^6^A regulate gene expression. **(A)** schematic representation of bi-directional regulation in co-transcriptional machinery. LncRNAs can change histone dynamics, while histones control lncRNA expression. M^6^A on lncRNAs modulate RNA metabolism, while lncRNAs guide m^6^A patterning. Finally, m^6^A alters histone modifications, while histone modifications pattern m^6^A modification. **(B)** Crosstalk between lncRNAs, histone modifications and m^6^A integrate distinct signals that alter upstream epigenetic landscape and downstream RNA metabolism.

Since the first observation that lncRNAs undergo m^6^A methylation (Meyer et al., 2012), a multitude of studies have expanded the repertoire and importance of m^6^A modified lncRNAs(Fazi and Fatica, 2019; Lv et al., 2020; Xue et al., 2020). Conversely, a few yet pivotal studies have identified role of lncRNAs in guiding the m^6^A writer complex, readers, and erasers to mRNA targets (Figure 3A). A particular example is that of the cis-acting lncRNA GATA3-AS and its ability to recruit VIRMA and facilitate the m^6^A modification of the 3′UTR of GATA3 pre-mRNA. The downstream effect of GATA3 m^6^A methylation was disrupted binding of HuR protein, down regulation of GATA3, and increased metastasis of liver cancer (Lan et al., 2019). More studies are necessary to elucidate the mechanism by which lncRNA recruits VIRMA and the structural changes induced by lncRNA-mRNA binding that would alter writer complex activity to pattern m^6^A.

M^6^A readers and erasers have been described to utilize both cis- and trans-acting lncRNAs as guides. LINC00857 was observed to cooperate with reader YTHDC1 to increase the stability of SLC7A5 mRNA in colorectal cancer cells (Tang et al., 2021). The lncRNA KB 1980E6.3 was found to form an RNA: protein complex with the m^6^A reader IGF2BP1 to facilitate the recognition and mRNA stability of m^6^A modified c-Myc in breast cancer stem cells (Zhu et al., 2021). LncRNAs have been found to interact with both m^6^A FTO and ALKBH5 Eraser proteins. FOXM1-AS increases the interaction of FOXM1 and ALKBH5, promoting demethylation of FOXM1 decreasing both FOXM1 expression and tumor growth (Zhang et al., 2017). In a similar study, the lncRNA GAS-AS1 was found to promote the ALKBH5-dependent demethylation of GAS mRNA and inhibit cervical cancer proliferation (Wang et al., 2019; Chen et al., 2020). Additionally, the lincRNA CASC15 is thought to recruit the demethylase FTO to SIM2, decreasing SIM2 mRNA stability and promoting esophageal cancer progression (Qin et al., 2020). Furthermore, specific lncRNAs such as CACNA1G-AS1 and ACAP2-IT1 have been predicted to regulate m^6^A readers and writers expression (Zheng et al., 2021). These initial studies provide substantial evidence that lncRNAs have dynamic interactions with m^6^A proteins, and additional research is likely to provide further examples.

### Chromatin Modifications and m^6^A Deposition

There is a growing body of literature that describes bi-directional interactions between the epigenome and the epitranscriptome (Figure 3A). This was first observed in the context of m^6^A methylation upon knock-down of m^6^A writer Mettl14, which altered the expression of histone modifying proteins (Y. Wang Z. et al., 2018). Since then, manipulations of readers, writers, and erasers, as well as the m^6^A modification itself, have been found to impact histone modifications. See Kan et al. for recent review (Kan et al., 2022). A clear example was the observation that m^6^A could co-transcriptionally direct the demethylation of histone H3K9me2 (Li et al., 2020)**.** This occurs by m^6^A reader YTHDC1 physically interacting with the H3K9me2 demethylase KDM3B at m^6^A-associated chromatin regions, promoting H3K9me2 demethylation and increasing overall gene expression. In another example, H3K27me3 was described as a barrier for m^6^A modification during transcription. Furthermore, the histone demethylase KDM6B that targets H3K27me3 directly recruits writers Mettl3 and Mettl14 to facilitate m^6^A methylation of co-transcribing mRNAs while simultaneously promoting transcription (Wu et al., 2020).

Recently, chromatin remodeling by H3K36me3 was observed to pattern m^6^A at the CDS and 3′UTR regions of RNA (Huang et al., 2019). Specifically, H3K36me3 scantly effected m^6^A levels in the 5′UTR in contrast to the CDS and 3′UTR. Furthermore, the repressive histone mark H3K9me3 was negatively correlated with m^6^A peaks, and metagene profiles of m^6^A at H3K36me3-negative sites correlated with increased 5′UTR methylation (Huang et al., 2019). Additionally, all the members of the core m^6^A writer complex, Mettl14, Mettl3 and WTAP, were found to bind with H3K36me3 and not with H3K9me3. However, members of the associated writer complex, VIRMA, Zc3h13, and Hakai were not tested. Interestingly, individual shRNA silencing of Mettl14, Mettl3 or WTAP did not dissociate the remaining m^6^A writer complex proteins from H3K36me3, which warrants future investigation.

As described, H3K36me3 peaks were anti-correlated with m^6^A at the 5′UTR (Huang et al., 2019). This discrepancy H3K36me3 relative to m^6^A patterning can be rationalized by considering the “histone code.” It is generally accepted that a gene is occupied by multiple nucleosomes, given that a nucleosome repeat consists of 140–200 bp of DNA. While the length of the mammalian 5′UTR can range between few nucleotides to several thousand, the median length of the 5′UTR in humans and mice is of 218 and 175, respectively (Leppek et al., 2018). Additionally, the first nucleosome immediately after the transcriptional start site (TSS), e.g., the one that may occupy the 5′UTR, exhibits distinct regulatory dynamics when compared to those of the CDS (Zhang and Pugh, 2011). These correlations warrant further exploration of how the epigenetic landscape patterns m^6^A on the 5′UTRs co-transcriptionally. Consequently, other histone post-translational modifications and the role of 3D DNA organization need to be explored in the context of m^6^A methylation.

### LncRNA Interacting With Chromatin Organizers

There is an extensive body of literature that describes lncRNAs interacting with the histone modifiers (Yao et al., 2019) (Figure 3A). Interestingly, lncRNA databases predict that at least 20% of lncRNAs guide DNA/protein and chromatin interactions within the nucleus (Volders et al., 2015). This is impressive, given over 10,000 have been predicted to exist (Volders et al., 2015). This account supports the abundant discovery of lncRNAs that interact with chromatin modifiers. This section reviews major findings of lncRNAs interacting with histone methylation proteins, as to highlight the potential of lncRNAs to interact with histone modifiers, enabling m^6^A patterning of mRNA transcripts.

As previously mentioned, H3K36me3 can guide m^6^A methylation co-transcriptionally (Huang et al., 2019). Multiple lncRNAs such as MEG3 (Terashima et al., 2017), Kcnq1ot1 (Pandey et al., 2008) and Air (Nagano et al., 2008) interact directly with histone methyltransferases for H3K36, and specifically regulate H3K36me3. LncRNAs have been found to interact with a variety of histone methyltransferases. An interesting example is that of HOTTIP, a divergently expressed lncRNA that promotes entire gene-expression programs by H3K4me3 patterning (Wang et al., 2011). In addition, the lncRNA Hotair that binds to G-A base pair rich DNA, correlates with H3K27me3 peaks (Chu et al., 2011). Deep-sequencing has also revealed both cis- and trans-acting lncRNAs, with 218 confirmed lincRNAs that bind directly with the Polycomb repressive complex 2 (PRC2), a protein complex that exhibits histone methyltransferase activity primarily on H3K27me3 (Zhao et al., 2010).

## Final Remarks

It is unlikely any specific pathway will be found to exclusively regulate m^6^A methylation patterns. This is perhaps due to the diversity of proteins within the writer complex contributing to a combinatorial mechanism to dictate m^6^A deposition. While lncRNAs may not be the exclusive mechanism that guides UTR m^6^A methylation, it is a contributor of m^6^A patterning in RNA, as it is for DNA and histones. A continuum of interesting phenomena hasbeen described to pattern the RNA modifications, and future research will likely describe these multiple mechanisms as cofactors in the crosstalk of the epigenome and the epitranscriptome (Figure 3B). Such findings will elucidate previously undescribed RNA interactions to which disease or single nucleotide polymorphisms (SNPs) may be attributed. Future research will provide more examples of extensive cross talk between the epigenome and epitranscriptome. Most likely positive and negative feedback systems, as well as sources of illness and targets of intervention.

## Glossary

ANRIL: antisense noncoding RNA in the INK4 locus
AS: anti-sense
AS-lncRNAs: anti sense long non-coding RNAs
Bp: base pairs
CDS: coding sequence
EMT: epithelial mesenchymal transition
FISH: fluorescent *in situ* hybridization
H2A: histone H2A
H2B: histone H2B
H3: histone H3
H3K4me1: histone H3 lysine 4 methylation
H3K9me2: histone H3 lysine 9 di-methylation
H3K27ac: histone H3 lysine 27 acetylation
H3K27me3: histone H3 lysine 27 tri-methylation
H3K36me3: histone H3 lysine 36 tri-methylation
H4: histone H4
HDAC2: histone deacetylase 2
**IRES** **-**: internal ribosome entry site
lincRNAs: large intergenic non-coding RNAs
lncRNA: long non-coding RNAs
m6A: N6-methyladenosine
miRNA: micro RNAs
mRNA: messenger RNA
ncRNA: non-coding RNA
ORFs: open-reading frames
PRC2: polycomb repressive complex 2
rRNAs: ribosomal RNAs
scaRNAs: cajal-body-associated RNAs
snoRNAs: small nucleolar RNAs
